# Cation Effects on Hydrogen Oxidation Reaction on Pt Single-Crystal Electrodes in Alkaline Media

**DOI:** 10.1021/acs.jpclett.4c00292

**Published:** 2024-03-07

**Authors:** Linfan Shen, Akansha Goyal, Xiaoting Chen, Marc T. M. Koper

**Affiliations:** † Leiden Institute of Chemistry, Leiden University, PO Box 9502, 2300 RA Leiden, The Netherlands; ‡ School of Materials Science and Engineering, Beijing Institute of Technology, Beijing 100081, P. R. China

## Abstract

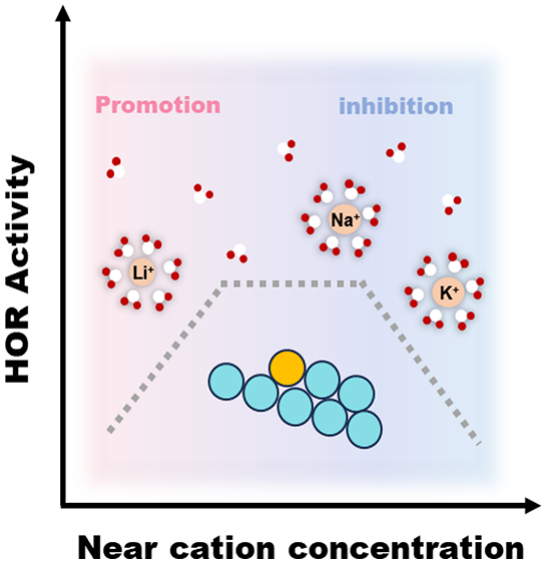

The exact mechanism behind the cation-assisted hydrogen oxidation reaction (HOR) on platinum electrodes in alkaline media remains disputed. We show that the cation effects at platinum display a remarkable structure sensitivity: not only the H adsorption but also the HOR activity on (111) terrace sites are independent of the nature of cation and cation concentration. On (110) step sites, at low cation concentration and mildly alkaline media, cations promote the HOR, whereas at more alkaline pH and consequently higher near-surface cation concentrations, the HOR is inhibition by the cations. Moreover, the role of the cation on terrace–OH_ad_ is different from that on step–OH_ad_, as can also be observed from the inhibition of the HOR current by terrace–OH_ad_ at higher potentials. These results suggest that near the onset potential, HOR mainly takes place on steps, but under diffusion-limited conditions at higher overpotential, HOR mainly takes place on terraces.

The hydrogen oxidation reaction (HOR) and its reverse reaction, the hydrogen evolution reaction (HER), are the most fundamental electrocatalytic reactions involving the simplest molecule H_2_, which is a key energy carrier and a very likely fuel of the future.^1,2^ Electrocatalysis takes place at the (solid) electrode–liquid electrolyte interface. Many previous studies focused on the chemical interactions of adsorbates with the electrode surface. For alkaline media, it has been proposed that both optimal hydrogen (H_ad_) and hydroxyl (OH_ad_) binding energetics are needed to achieve the best activity.^3,4^ More recently, with the proposals that noncovalent interactions and the reorganization of interfacial water impact the rate of electrocatalytic reactions, the importance of processes on the electrolyte side of the interface has been put forward.^5−7^ Alkali metal cations are one of the important electrolyte parameters in describing the hydrogen electrode reaction kinetics in alkaline media, as they can have a strong interaction with the metal surface as well as with the interfacial water and OH_ad_. However, the exact mechanism behind the cation-assisted HOR and HER in alkaline media remains disputed.^8−10^

Our group has recently investigated the cation effect on HER, showing that the HER kinetics on polycrystalline Pt improves in going from K^+^ < Na^+^ < Li^+^, in agreement with previous results.^11−14^ Remarkably, on single-crystalline Pt, we showed that the cation effect is strictly limited to step sites; the effect of cations on the HER rate on two-dimensional (111) and (100) facets is much less notable.^15^ However, whether these cation effects on the rate of the HOR are equal to those of the HER is not well documented.^16,17^ Therefore, in this article, we use Pt(111)-type single-crystal electrodes to systemically study the relation between the cation effect and structural order of Pt on the rate of HOR in alkaline media. Largely mirroring the trends for HER, the HOR activity is not affected by the nature of cation or the cation concentration on Pt(111), while the HOR kinetics improves in going from K^+^ < Na^+^ < Li^+^ on Pt(553) and Pt(110). It reveals how surface structure and surface charge distribution influence the efficiency of catalytic reactions, which is crucial for understanding and improving catalyst design.

In Figure 1, we compare the blank voltammograms and HOR activity at three Pt single-crystal electrodes, i.e., Pt(111), Pt(553), and Pt(110), in 0.1 M alkaline electrolyte containing either Li^+^, Na^+^ or K^+^ cations. As shown in Figures 1a and 1d, for Pt(111), in the H_upd_/*H region (0.05 V_RHE_ < *E* < 0.4 V_RHE_), both the hydrogen adsorption feature and the HOR activity remain unaffected by the nature of the cation. The same is true for the HER activity below 0 V, in agreement with our previous observation that on Pt(111) the HER activity is independent of cation identity and concentration.^15^ The situation is drastically different for surfaces containing (110) step sites, i.e., Pt(553) and Pt(110). In the blank CV (Figures 1b and 1c), there is a step-related voltammetric peak (around 0.25 V) which involves the replacement of *H by *OH.^18,19^ The step-related peak shifts to more positive potentials with increasing (unsolvated) alkali cation size, which has been explained by the competition between *OH and near-surface cations for solvating water molecules.^19^ The implication of this interpretation of the step-related peak is that *OH is formed at steps at low potentials, basically from ca. 0.25 V. The HOR activity improves with smaller (unsolvated) cation size, i.e., K^+^ < Na^+^ < Li^+^, on Pt surfaces with (110) step sites (Figures 1e and 1f). The cation identity trend is independent of step density and identical for the HER, as can also be observed for the HER currents in Figures 1e and 1f. These data clearly illustrate that our previous conclusions for the cation-assisted HER on Pt also hold for HOR: the cation effect is limited to step sites (i.e., it does not take place on (111) terraces), and Li^+^ shows the highest promoting effect. Note, however, that this cation promotion effect on HOR/HER happens at potentials negative of the step-related peak, and hence there is no clear (voltammetric) evidence for the involvement of step-adsorbed OH in this promotion effect.

**Figure 1:**
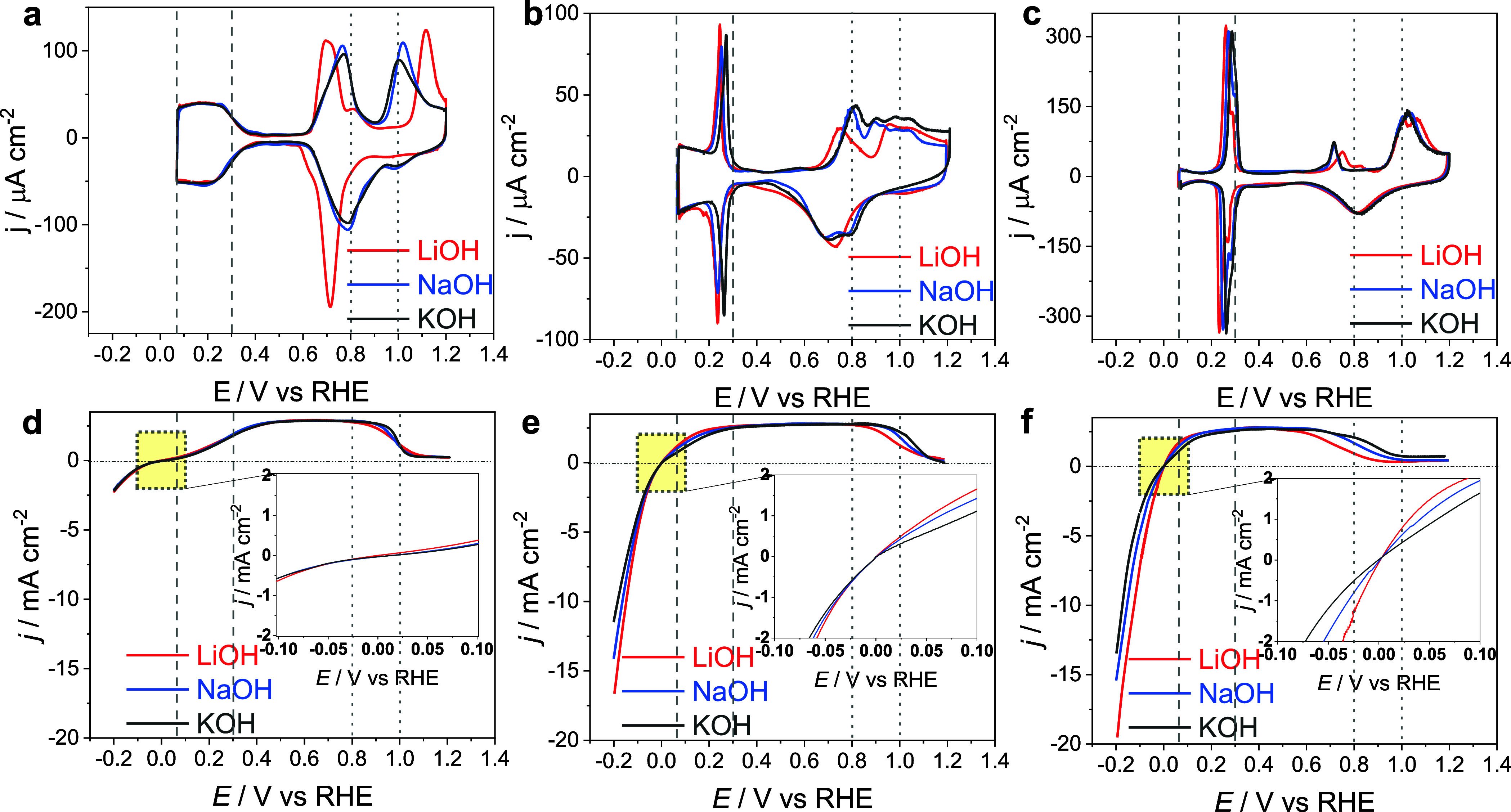
Cyclic voltammograms of (a) Pt(111), (b) Pt(553), and (c) Pt(110) in 0.1 M MeOH. HOR activity of (d) Pt(111), (e) Pt(553), and (f) Pt(110) in 0.1 M MeOH, where Me is Li, Na, or K. All experiments at rotation rates of 1600 rpm and a sweep rate of 10 mV/s.

The most likely interpretation for this observed step-related cation promotion is that cations interact more strongly with the (110) step sites than with the (111) terrace sites, in agreement with capacitance measurements presented in our previous work.^15^ We explain the cation identity trend for the HOR kinetics on the basis of the changes in the cation solvation energy and how these changes correlate with the interactions of the cations with the metal interface and the reacting water molecule.^16^ Our previous results have shown that weakly hydrated cations stabilize the transition state of the water dissociation step more favorably.^17^ Based on these results, we propose that HOR kinetics improve with increasing step density and decreasing solvation energy. Moreover, the Tafel slope of ∼120 mV dec^–1^ (on Pt(111)) further indicates that the first electron transfer step is rate determining (Figure S1). Cations near the surface can enhance the HOR activity on Pt electrodes by favorably interacting with the transition state of the rate determining Volmer step (*H + OH^–^ + cat^+^ → *H--OH_step_^δ−^--cat^+^ + (1 – δ)e^–^→ H_2_O + e^–^ + ∗ + cat^+^).

Interestingly, there is also a cation effect on the terrace–OH_ads_ region (0.65 < *E* < 0.85 V) with a corresponding drop in HOR current in the same potential window. This drop in current density must be related to the formation of adsorbed OH (*OH), as can be inferred from the blank CV. As has been observed before,^7,20^ the formation of *OH is sensitive to the nature of the electrolyte cation. The *OH peak appears at a lower potential in the Li^+^-containing electrolyte (0.70 V) compared to Na^+^- and K^+^-containing electrolyte (0.77 V). Correspondingly, the HOR current density drops at a (slightly) lower potential in Li^+^-containing electrolytes. As on Pt(111), the onset of the OH adlayer formation on the (111) terrace of Pt(553) in the presence of Li^+^ is shifted to more negative potentials by about 20 mV compared to the other cations.^18^ The drop in HOR current follows the same trend as the shift in *OH adlayer formation, suggesting that the formation of the *OH adlayer on (111) terraces is responsible for the blocking of the HOR at high potentials (note again that *OH on the steps should have already formed at ca. 0.25 V). In the terrace–OH_ads_ region, Li cations stabilize the OH layer the most, which leads to the highest OH coverage, a corresponding decrease in the number of reactive platinum sites, and a subsequent decrease in the HOR reaction rate. The situation is a bit less clear (and reproducible) for Pt(110): the drop in HOR current density happens at lower potentials than on Pt(111) and Pt(553), although the trend with cation size is the same. It is important to note that the actual surface structure of Pt(110) is not nominal: it has a strong tendency to reconstruct, and the extent of the reconstruction is sensitive to various parameters.^21−23^ From these results, we conclude that at these high overpotentials, leading to diffusion-limited conditions, HOR takes place (only) on the (111) terraces. Once they are blocked, the HOR decreases to very low values.

The interesting ramification of this conclusion is that the nature of the active site changes with potential: close to the equilibrium potential, the step sites are the most active, whereas at a more positive potential, the (111) terrace sites are the active sites (and they are now active enough to sustain a mass-transport-limited current). Presumably, the step sites have become inactive at an intermediate potential, likely due to the adsorption of *OH in the step-related peak at 0.25 V.^19^

We have performed the same experiments at pH 11, as our previous experiments on HER showed that there is a principal difference in the effect of cations between pH 11 and 13.^15^ For pH 11, two mass-transfer-dependent plateaus are observed, indicating that the overall current is influenced by two separate processes (species). We will refer to the diffusion-limiting current at lower potential as the first plateau and to the one at more positive potential as the second plateau. As shown in Figure S2, the current density of the first plateau is proportional to the OH^–^ concentration. According to the Levich equation (eq 1), the diffusion limiting current (*j*_lim_) is
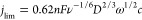
1where *n* is the number of electrons transferred, *F* is the Faraday constant, *v* is the kinematic viscosity of water, *D* is the diffusion coefficient, ω is the rotation rate, and *c* is the bulk concentration of the diffusing species.^24^ From the Levich plots for the first and second current plateaus, the diffusion coefficient can be calculated using the slope of the linear fit of 1/*j*_lim_ vs the inverse of the square root of the rotation rate (if the relevant bulk concentrations are known). The average derived diffusion coefficients are 4.4 × 10^–5^ and 8.3 × 10^–5^ cm^2^ s^–1^ for the first and second limiting currents, using the concentration of OH^–^ and H_2_ as the parameter *c* in eq 1 (Figure S3).^25,26^ The comparison of the experimentally obtained and tabulated diffusion coefficients indeed indicates that the first plateau current is governed by the mass transport of OH^–^ to the surface, while the second plateau is determined by the mass transport of H_2_ to the surface, as shown in eqs 2 and 3:
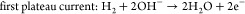
2
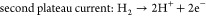
3This identification agrees with the cation dependence of the first and second plateaus. As expected, the reaction leading to the first plateau is cation dependent on Pt(553) and Pt(110), but not on Pt(111), while the process leading to the second plateau is cation independent of all surfaces (Figure 2a–c).

**Figure 2:**
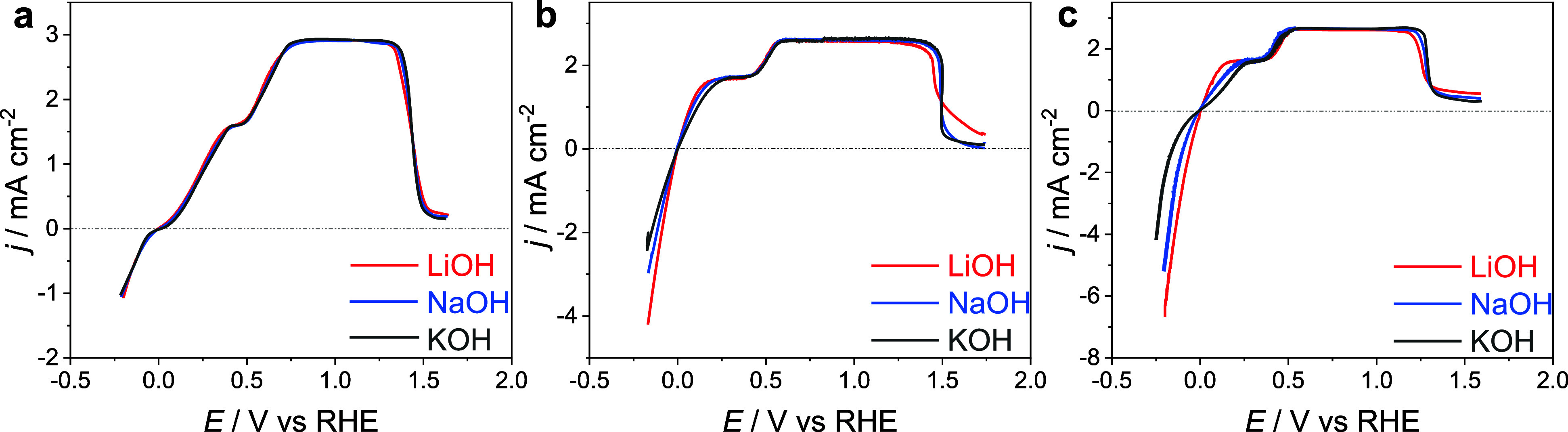
HOR curves of (a) Pt(111), (b) Pt(553), and (c) Pt(110) in 0.001 M MeOH + 0.099 M MeClO_4_, where Me is Li, Na, or K, as indicated. The HOR test was performed at rotational speeds of 1600 rpm and a sweep rate of 10 mV/s.

To further illustrate the cation effects, we studied the effect of cation concentration on the kinetics of HOR for bulk electrolytes pH 11 and 13 on the three different surfaces. Figures S4–S6 show the blank CVs under the different conditions, and Figures S7–S10 depict the HOR cyclic voltammetry at various cation concentrations. As shown in Figure 3, for Na^+^-containing electrolytes (see Figures S10 and S11 for Li^+^- and K^+^-containing electrolytes), the cation reaction order on the Pt(111) terrace at pH 11 and 13 is almost ∼0, in agreement with previous observations for HER.^15^ On Pt(553) and Pt(110), we obtain negative reaction orders in cation concentration at pH 13, with K^+^ exhibiting a larger negative reaction order than Li^+^. At pH 11, we observe positive reaction orders in cation concentration increasing from Li^+^ < Na^+^ < K^+^. These results indicate that as the electrolyte pH, and thus, the near surface cation concentration increase, above a threshold (saturation) concentration, the promotional effect of the cations on the HOR kinetics disappears and, at extremely high cation concentrations, becomes inhibitive, very similar to what has been observed for HER.^11^

**Figure 3:**
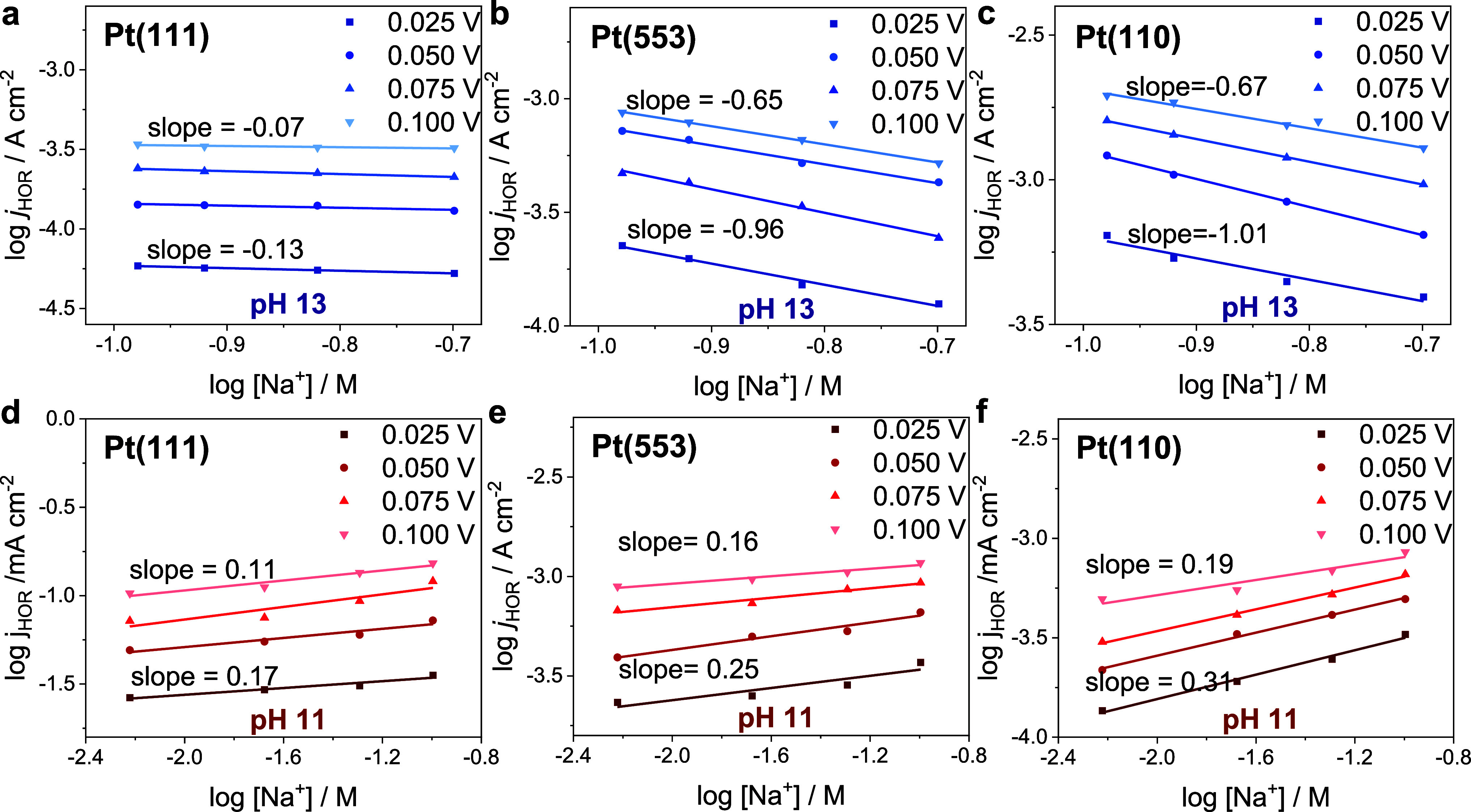
Reaction order plots obtained for HOR on (a) Pt(111), (b) Pt(553), and (c) Pt(110) at pH 13 and in (d) Pt(111), (e) Pt(553), and (f) Pt(110) at pH 11 (NaOH) with varying cation concentration in the bulk, at 1600 rpm and at a scan rate of 10 mV s^–1^. The slope indicates the corresponding cation reaction order at a fixed potential (vs RHE).

Interestingly, the reaction orders for all the cations are smaller than 1, indicating that the inner-layer cation concentration is approaching saturation already at intermediate pH values. Thus, at high cation concentrations, the promotional effect of cations reaches a plateau and subsequently becomes inhibitive, which we ascribe to the crowding of the reactive surface by near-surface cations. Whether cations chemically adsorb at the surface or just collect in the outer Helmholtz plane in the double layer remains a contentious issue. In either case, it would be expected that the accumulated cations near the surface can result in detrimental effects for HOR if the cation–metal interactions are boosted at the expense of water–metal interactions. This hypothesis also explains the stronger “blocking” effect at pH 13 in K^+^ ion containing electrolytes compared to Li^+^ ion containing electrolytes because Li^+^ cations have the weakest interaction with the metal surface due to their higher degree of solvation (Figures S11 and S12). Hence, these results suggest that intermediate electrolyte pH is optimal for the cation-assisted HOR mechanism in alkaline media, and a more extreme near-surface cation concentration at very high pH can lead to a decrease in HOR activity as the strong cation–metal interactions render the metal surface less active for HOR. Similar trends in the experimental reaction orders for the cation concentration and the electrolyte pH suggest that these two factors are regulating the same active species at the interface.

In conclusion, we investigated the cation effects on the HOR on the stepped Pt(111)-type single crystal electrodes in alkaline solution. We discovered that the cation effect on HOR is largely mirroring the trend of HER. The cation effect on stepped Pt(111)-type single crystals displays a remarkable structural sensitivity: no cation concentration and identity effects were observed at Pt(111) terrace in the H_upd_ region. In contrast, the HOR kinetics on step sites is improved in going from K^+^ < Na^+^ < Li^+^ (at pH 13); this can be understood as inhibition of the Volmer step by a weakly hydrated cation. At pH 11, there is a promotion effect with increasing cation concentration, mirroring the pH-dependent cation effect observed previously for HER on polycrystalline and single-crystalline Pt and Au.^11,13,15^ At high overpotentials, cations also affect the HOR inhibition. Because the inhibition follows the (cation-dependent) OH formation on (111) terraces, this suggests that at high overpotential HOR primarily takes place on the (111) terraces.

## Supporting Information Available

Further experimental details and methods, polarization curves and voltammograms, Tafel plots, Levich plots, and reaction-order plot (PDF)

## References

[ref1] Prats H.; Chan K. The Determination of the HOR/HER Reaction Mechanism from Experimental Kinetic Data. Phys. Chem. Chem. Phys. 2021, 23 (48), 27150–27158. 10.1039/D1CP04134G. 34852033

[ref2] Rebollar L.; Intikhab S.; Oliveira N. J.; Yan Y.; Xu B.; McCrum I. T.; Snyder J. D.; Tang M. H. Beyond Adsorption” Descriptors in Hydrogen Electrocatalysis. ACS Catal. 2020, 10 (24), 14747–14762. 10.1021/acscatal.0c03801.

[ref3] Sheng W.; Myint M.; Chen J. G.; Yan Y. Correlating the Hydrogen Evolution Reaction Activity in Alkaline Electrolytes with the Hydrogen Binding Energy on Monometallic Surfaces. Energy Environ. Sci. 2013, 6 (5), 1509–1512. 10.1039/c3ee00045a.

[ref4] Strmcnik D.; Uchimura M.; Wang C.; Subbaraman R.; Danilovic N.; van der Vliet D.; Paulikas A. P.; Stamenkovic V. R.; Markovic N. M. Improving the Hydrogen Oxidation Reaction Rate by Promotion of Hydroxyl Adsorption. Nat. Chem. 2013, 5 (4), 300–306. 10.1038/nchem.1574. 23511418

[ref5] Ledezma-Yanez I.; Wallace W. D. Z.; Sebastián-Pascual P.; Climent V.; Feliu J. M.; Koper M. T. M. Interfacial Water Reorganization as a pH-Dependent Descriptor of the Hydrogen Evolution Rate on Platinum Electrodes. Nat. Energy 2017, 2 (4), 17031 10.1038/nenergy.2017.31.

[ref6] Danilovic N.; Subbaraman R.; Strmcnik D.; Paulikas A. P.; Myers D.; Stamenkovic V. R.; Markovic N. M. The Effect of Noncovalent Interactions on the HOR, ORR, and HER on Ru, Ir, and Ru_0.50_Ir_0.50_ Metal Surfaces in Alkaline Environments. Electrocatalysis 2012, 3 (3), 221–229. 10.1007/s12678-012-0100-7.

[ref7] Strmcnik D.; Kodama K.; van der Vliet D.; Greeley J.; Stamenkovic V. R.; Marković N. M. The Role of Non-Covalent Interactions in Electrocatalytic Fuel-Cell Reactions on Platinum. Nat. Chem. 2009, 1 (6), 466–472. 10.1038/nchem.330. 21378914

[ref8] Huang B.; Rao R. R.; You S.; Hpone Myint K.; Song Y.; Wang Y.; Ding W.; Giordano L.; Zhang Y.; Wang T.; Muy S.; Katayama Y.; Grossman J. C.; Willard A. P.; Xu K.; Jiang Y.; Shao-Horn Y. Cation- and pH-Dependent Hydrogen Evolution and Oxidation Reaction Kinetics. JACS Au 2021, 1 (10), 1674–1687. 10.1021/jacsau.1c00281. 34723270 PMC8549054

[ref9] Waegele M. M.; Gunathunge C. M.; Li J.; Li X. How Cations Affect the Electric Double Layer and the Rates and Selectivity of Electrocatalytic Processes. J. Chem. Phys. 2019, 151 (16), 160902 10.1063/1.5124878. 31675864

[ref10] Rebollar L.; Intikhab S.; Snyder J. D.; Tang M. H. Determining the Viability of Hydroxide-Mediated Bifunctional HER/HOR Mechanisms through Single-Crystal Voltammetry and Microkinetic Modeling. J. Electrochem. Soc. 2018, 165 (15), J3209–J3221. 10.1149/2.0271815jes.

[ref11] Monteiro M. C. O.; Goyal A.; Moerland P.; Koper M. T. M. Understanding Cation Trends for Hydrogen Evolution on Platinum and Gold Electrodes in Alkaline Media. ACS Catal. 2021, 11 (23), 14328–14335. 10.1021/acscatal.1c04268. 34888121 PMC8650008

[ref12] Goyal A.; Koper M. T. M. Understanding the Role of Mass Transport in Tuning the Hydrogen Evolution Kinetics on Gold in Alkaline Media. J. Chem. Phys. 2021, 155 (13), 134705 10.1063/5.0064330. 34624997

[ref13] Goyal A.; Koper M. T. M. The Interrelated Effect of Cations and Electrolyte pH on the Hydrogen Evolution Reaction on Gold Electrodes in Alkaline Media. Angew. Chem., Int. Ed. 2021, 60 (24), 13452–13462. 10.1002/anie.202102803. PMC8252582 33769646

[ref14] Xue S.; Garlyyev B.; Watzele S.; Liang Y.; Fichtner J.; Pohl M. D.; Bandarenka A. S. Influence of Alkali Metal Cations on the Hydrogen Evolution Reaction Activity of Pt, Ir, Au, and Ag Electrodes in Alkaline Electrolytes. ChemElectroChem. 2018, 5 (17), 2326–2329. 10.1002/celc.201800690.

[ref15] Goyal A.; Louisia S.; Moerland P.; Koper M. T. M. Cooperative Effect of Cations and Catalyst Structure in Tuning Alkaline Hydrogen Evolution on Pt electrodes. J. Am. Chem. Soc. 2024, 10.1021/jacs.3c11866. PMC10958517 38451209

[ref16] Liu E.; Li J.; Jiao L.; Doan H. T. T.; Liu Z.; Zhao Z.; Huang Y.; Abraham K. M.; Mukerjee S.; Jia Q. Unifying the Hydrogen Evolution and Oxidation Reactions Kinetics in Base by Identifying the Catalytic Roles of Hydroxyl-Water-Cation Adducts. J. Am. Chem. Soc. 2019, 141 (7), 3232–3239. 10.1021/jacs.8b13228. 30673227

[ref17] Shen L.f.; Lu B.A.; Qu X.m.; Ye J.y.; Zhang J.m.; Yin S.-h.; Wu Q.h.; Wang R.x.; Shen S.y.; Sheng T.; Jiang Y.x.; Sun S.g. Does the oxophilic effect serve the same role for hydrogen evolution/oxidation reaction in alkaline media?. Nano Energy 2019, 62, 601–609. 10.1016/j.nanoen.2019.05.045.

[ref18] van der Niet M. J. T. C.; Garcia-Araez N.; Hernández J.; Feliu J. M.; Koper M. T. M. Water Dissociation on Well-Defined Platinum Surfaces: The Electrochemical Perspective. Catal. Today 2013, 202, 105–113. 10.1016/j.cattod.2012.04.059.

[ref19] Chen X.; McCrum I. T.; Schwarz K. A.; Janik M. J.; Koper M. T. M. Co-adsorption of Cations as the Cause of the Apparent pH Dependence of Hydrogen Adsorption on a Stepped Platinum Single-Crystal Electrode. Angew. Chem., Int. Ed. 2017, 56 (47), 15025–15029. 10.1002/anie.201709455. PMC5991472 28987066

[ref20] Stoffelsma C.; Rodriguez P.; Garcia G.; Garcia-Araez N.; Strmcnik D.; Marković N. M.; Koper M. T. M. Promotion of the Oxidation of Carbon Monoxide at Stepped Platinum Single-Crystal Electrodes in Alkaline Media by Lithium and Beryllium Cations. J. Am. Chem. Soc. 2010, 132 (45), 16127–16133. 10.1021/ja106389k. 20979396

[ref21] Souza-Garcia J.; Climent V.; Feliu J. M. Voltammetric Characterization of Stepped Platinum Single Crystal Surfaces Vicinal to the (110) Pole. Electrochem. Commun. 2009, 11 (7), 1515–1518. 10.1016/j.elecom.2009.05.044.

[ref22] Lucas C. A.; Marković N. M.; Ross P. N. Surface Structure and Relaxation at the Pt(110)/Electrolyte Interface. Phys. Rev. Lett. 1996, 77 (24), 4922–4925. 10.1103/PhysRevLett.77.4922. 10062669

[ref23] Fery P.; Moritz W.; Wolf D. Structure Determination of the (1 × 2) and (1 × 3) Reconstructions of Pt(110) by Low-Energy Electron Diffraction. Phys. Rev. B 1988, 38 (11), 7275–7286. 10.1103/PhysRevB.38.7275. 9945449

[ref24] Marcandalli G.; Goyal A.; Koper M. T. M. Electrolyte Effects on the Faradaic Efficiency of CO_2_ Reduction to CO on a Gold Electrode. ACS Catal. 2021, 11 (9), 4936–4945. 10.1021/acscatal.1c00272. 34055454 PMC8154322

[ref25] Marcandalli G.; Monteiro M. C. O.; Goyal A.; Koper M. T. M. Electrolyte Effects on CO_2_ Electrochemical Reduction to CO. Acc. Chem. Res. 2022, 55 (14), 1900–1911. 10.1021/acs.accounts.2c00080. 35772054 PMC9301915

[ref26] Marcandalli G.; Villalba M.; Koper M. T. M. The Importance of Acid–Base Equilibria in Bicarbonate Electrolytes for CO_2_ Electrochemical Reduction and CO Reoxidation Studied on Au(hkl) Electrodes. Langmuir 2021, 37 (18), 5707–5716. 10.1021/acs.langmuir.1c00703. 33913319 PMC8154874

