# Supplementary material for: Cation Effects on Hydrogen Oxidation Reaction on Pt Single-Crystal Electrodes in Alkaline Media
Source: J Phys Chem Lett. 2024 Mar 7;15(10):2911–5. doi: 10.1021/acs.jpclett.4c00292 (PMC10945570; doi:10.1021/acs.jpclett.4c00292)
Supplement: Supplementary file 1 — jz4c00292_si_001.pdf [file jz4c00292_si_001.pdf]

Supporting information

Cation Effects on Hydrogen Oxidation Reaction on Pt Single Crystal Electrodes in Alkaline Media

Linfan Shen[1)], Akansha Goyal[1)], Xiaoting Chen[1,2)],

Marc T. M. Koper[1)*]

[1)] Leiden Institute of Chemistry, Leiden University, PO Box 9502, 2300 RA Leiden, the Netherlands

[2)] School of Materials Science and Engineering, Beijing Institute of Technology, Beijing 100081, P. R. China

Experimental methods

Chemicals

The electrolytes were prepared from LiOH (99.995%, Alfa Aesar), NaOH (32% by wt. solution, analysis grade, Merck), KOH (≥ 99.995%, Suprapur, Merck), $LiClO_4$ (99%, anhydrous, Alfa Aesar), $NaClO_4$ (99.99%, trace metals basis, Sigma-Aldrich), $KClO_4$ (≥ 99.99%, trace metals basis, Sigma-Aldrich), and Ultrapure water (MilliQ gradient, ≥18.2 MΩcm, TOC < 5 ppb). Ar (6.0 purity, Linde) and H_2 (5.0 purity, Linde) were used for purging the electrolytes.

General electrochemical methods

Cyclic voltammetric measurements were carried out in standard one-compartment electrochemical cells using a three-electrode assembly at room temperature. Experiments were performed in a fluorinated ethylene propylene (PEP, Nalgene1) electrochemical cell for alkaline solutions, whereas a glass cell was used for acidic electrolytes. All glassware was cleaned in an acidic solution of potassium permanganate overnight, followed by rinsing with an acidic solution of hydrogen peroxide and repetitive rinsing and boiling with ultrapure water. Pt(111), Pt(553), and Pt(110) with a diameter of 6.0 mm, 6.0 mm, and 4mm connected to platinum wire was used as the working electrode. Prior to every experiment, the working electrode was prepared according to the Clavilier method. A platinum wire was used as counter electrode and a reversible hydrogen electrode (RHE) was employed as the reference electrode, in a separate compartment filled with the same electrolyte, at the same pH as the electrolyte in the electrochemical cell. The electrochemical measurements were performed with the single-crystal electrode in the hanging meniscus configuration. The potential was controlled with an Autolab PGSTAT302N potentiostat. The resistance of the cell was determined before every experiment and the ohmic drop was

compensated accordingly. The pH value of the electrolyte was measured before every experiment by a pH meter. During the measurements, the glass pH electrode (Ag/AgCl) combined with the reference electrode (reversible hydrogen electrode) were applied to determine the pH value of the electrolyte. The current density shown in the manuscript represents the measured current normalized to the geometric area of the working electrode.

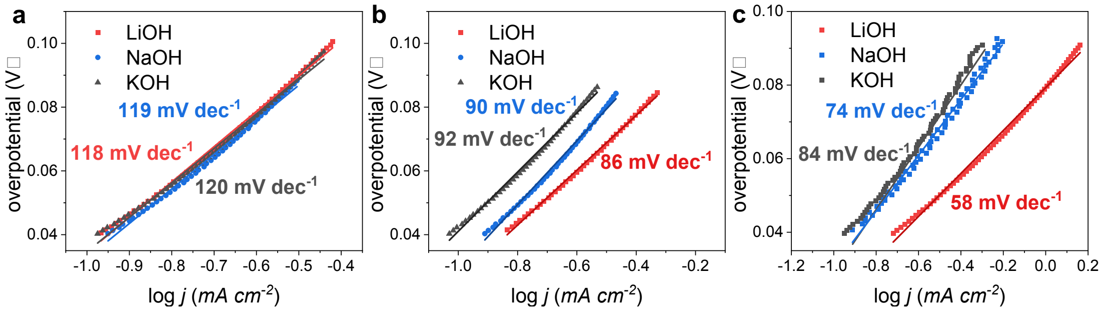

Figure S1. Tafel slopes on (a) Pt(111) , (b) Pt(553) and (c) Pt(110) in alkaline media (0.1 M MOH, pH = 13). M = Li^+, Na^+, and K^+.

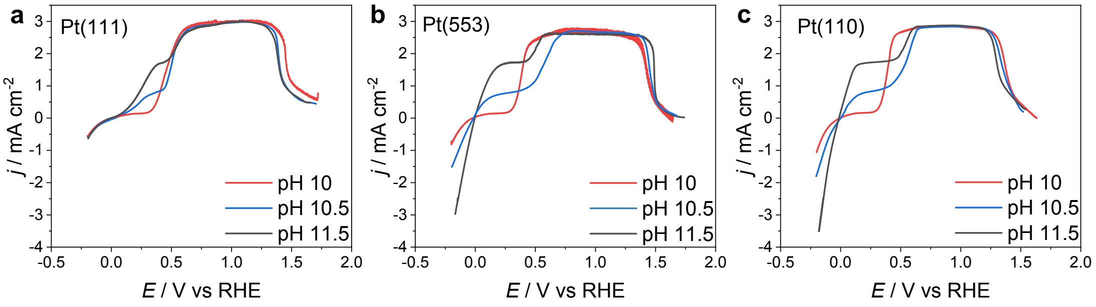

Figure S2. Polarization curves for the HER and HOR of (a) Pt(111), (b) Pt(553), and (c) Pt(110) in pH 10, 10.5 and 11.5 in NaOH electrolyte.

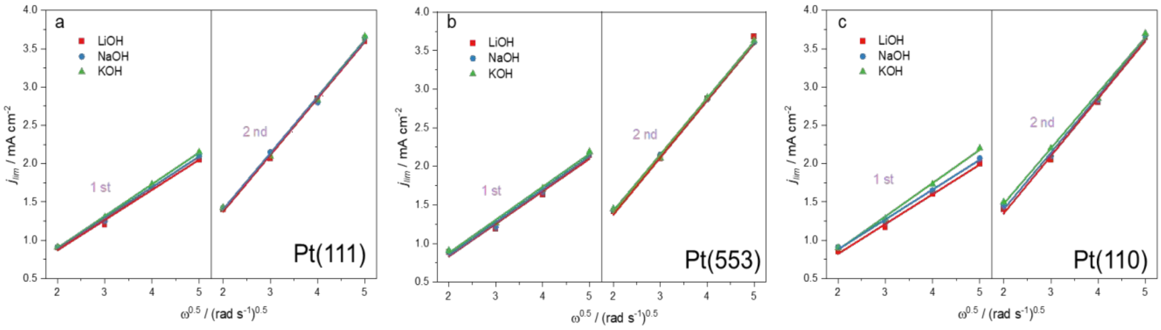

Figure S3. The Levich plots for (a) Pt(111), (b) Pt(553), and (c) Pt(110) in 0.001 M MeOH, where Me is Li, Na, or K, as indicated.

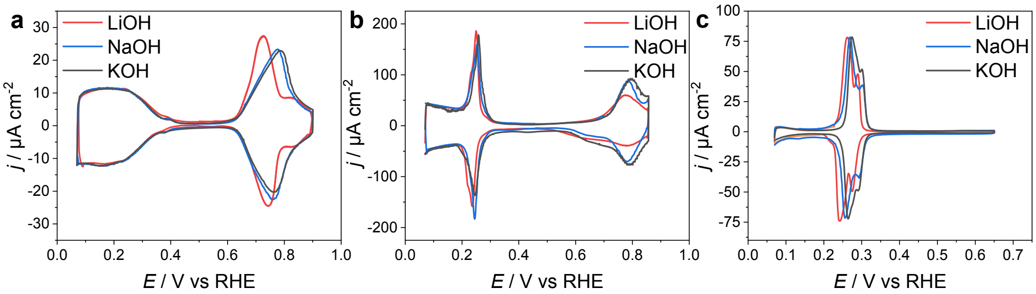

Figure S4. Cyclic voltammograms of (a) Pt(111), (b) Pt(553), and Pt(110) in 0.001 M MeOH + 0.099 M $MeClO_4$, where Me is Li, Na, or K, as indicated.

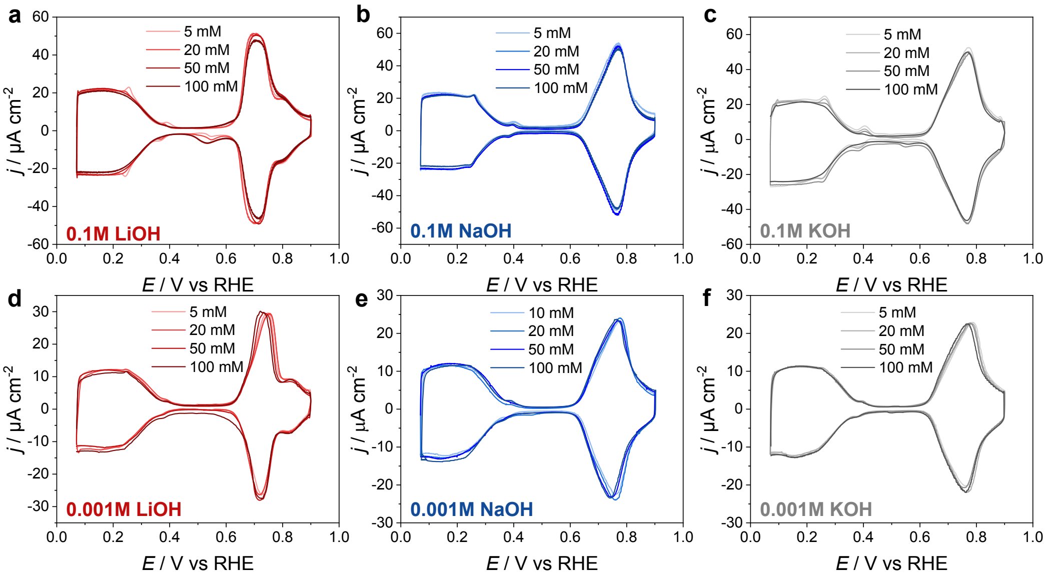

Figure S5. Cyclic voltammograms obtained on Pt(111) in 0.1M (a) LiOH, (b) NaOH, and (c) KOH, as well as 0.001 M (d) LiOH, (e) NaOH, and (f) KOH for different concentrations of corresponding $MeLiClO_4$ (5 mM, 20 mM, 50 mM and 100 mM), where Me is Li, Na, or K, as indicated.

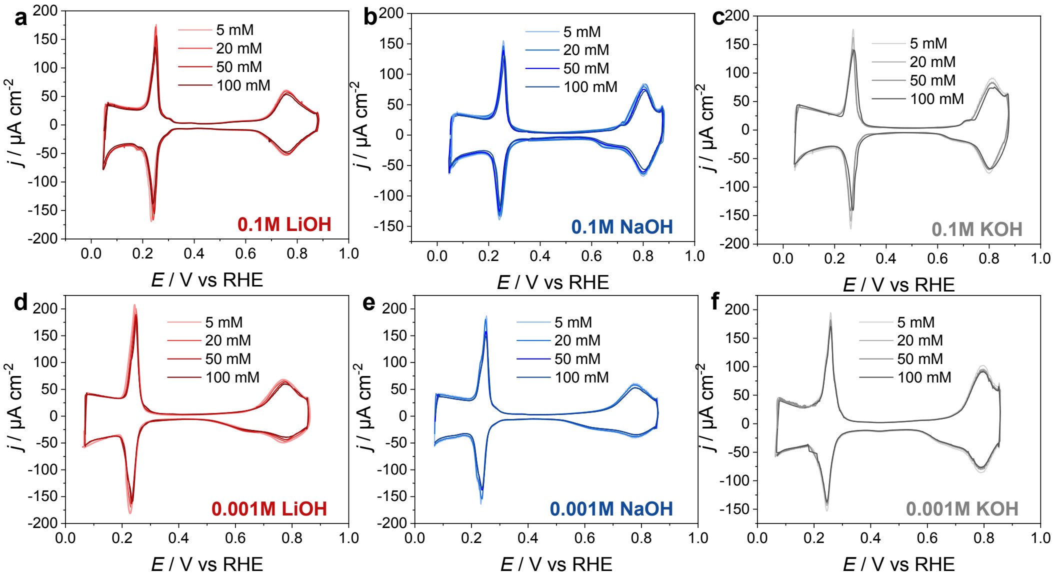

Figure S6. Cyclic voltammograms obtained on Pt(553) in 0.1M (a) LiOH, (b)NaOH, and (c) KOH, as well as 0.001 M (d) LiOH, (e)NaOH, and (f) KOH for different concentrations of corresponding $MeLiClO_4$ (5 mM, 20 mM, 50 mM and 100mM), where Me is Li, Na, or K, as indicated.

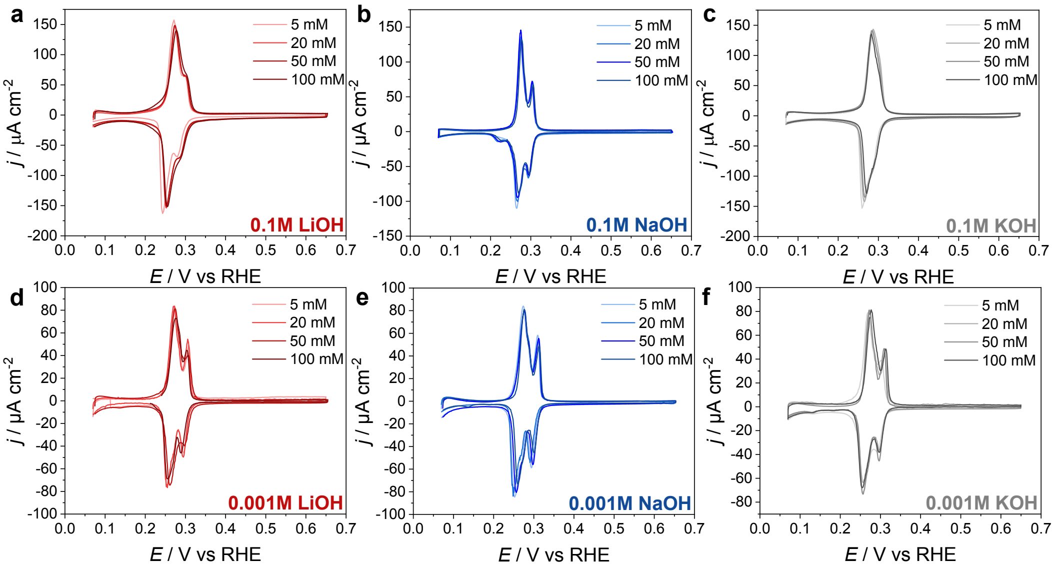

Figure S7. Cyclic voltammograms obtained on Pt(110) in 0.1M (a) LiOH, (b)NaOH, and (c) KOH, as well as 0.001 M (d) LiOH, (e) NaOH, and (f) KOH for different concentrations of corresponding $MeLiClO_4$ (5 mM, 20 mM, 50 mM and 100 mM), where Me is Li, Na, or K, as indicated.

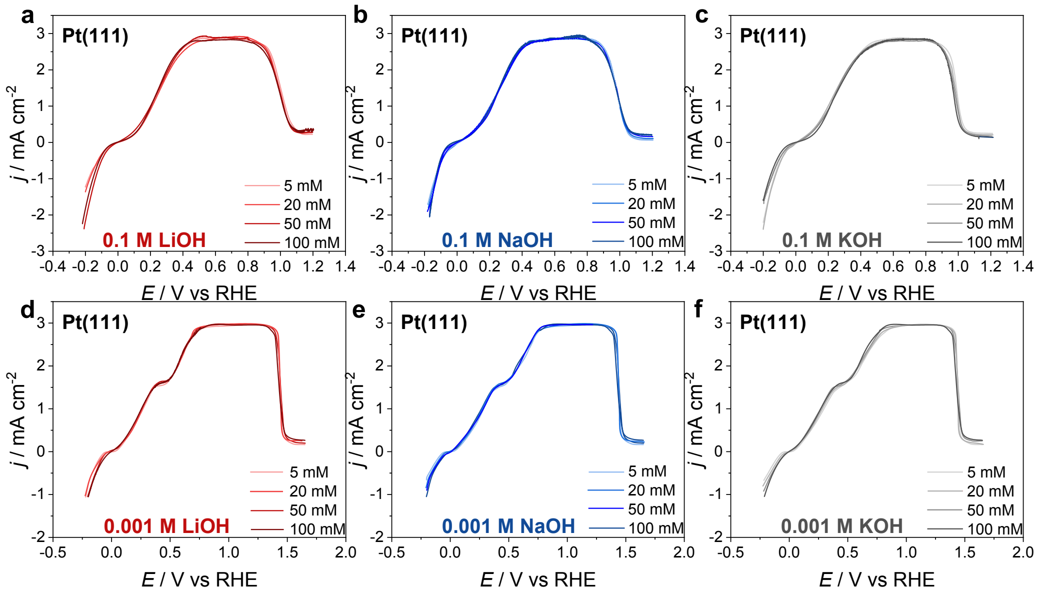

Figure S8. HOR curves obtained on Pt(111) in 0.1M (a) LiOH, (b)NaOH, and (c) KOH, as well as 0.001 M (d) LiOH, (e) NaOH, and (f) KOH for different concentrations of corresponding $MeLiClO_4$ (5 mM, 20 mM, 50 mM and 100 mM), where Me is Li, Na, or K, as indicated.

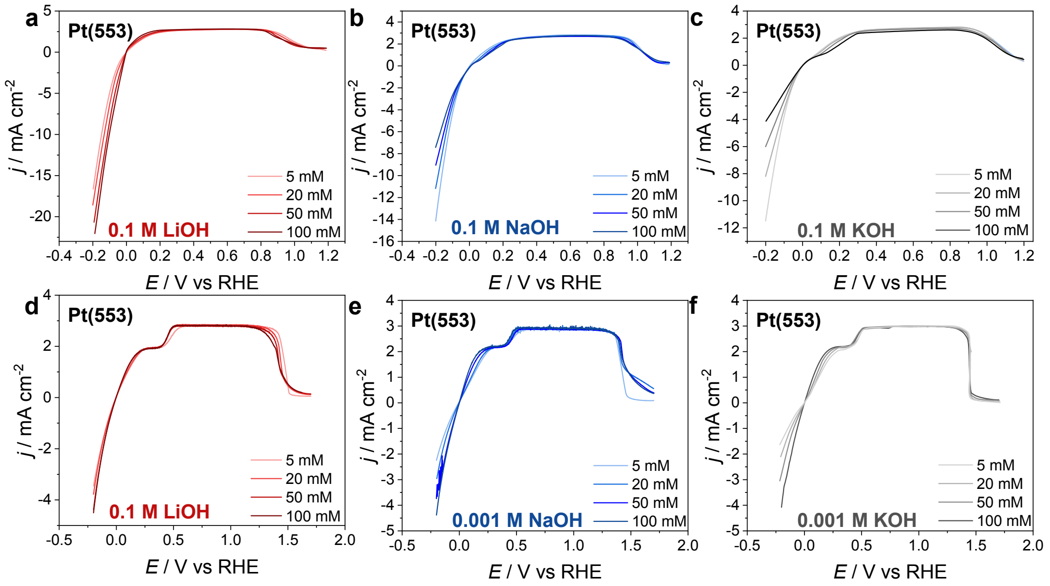

Figure S9. HOR curves obtained on Pt(553) in 0.1M (a) LiOH, (b)NaOH, and (c) KOH, as well as 0.001 M (d) LiOH, (e) NaOH, and (f) KOH for different concentrations of corresponding $MeLiClO_4$ (5 mM, 20 mM, 50 mM and 100 mM), where Me is Li, Na, or K, as indicated.

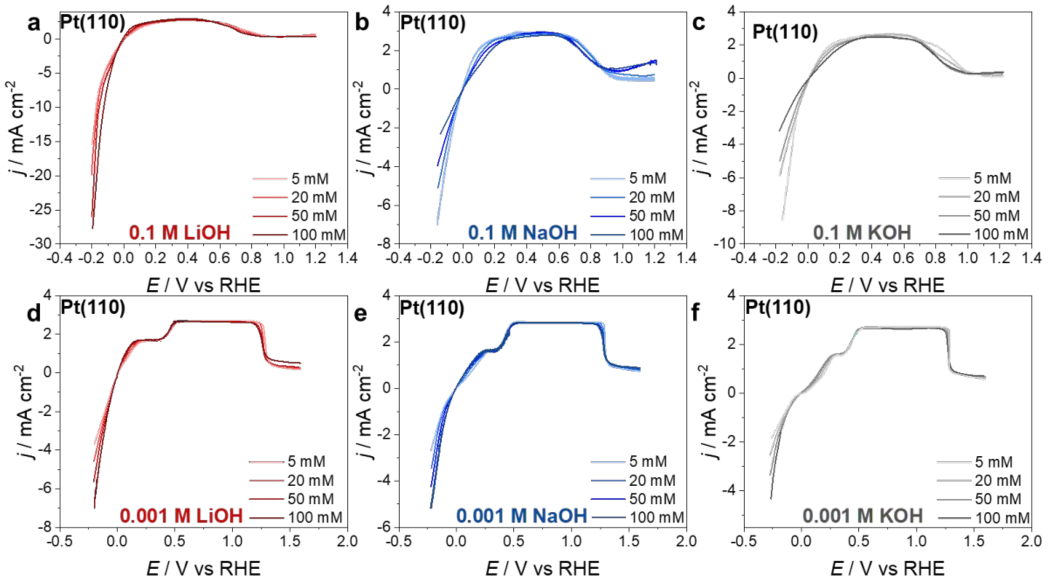

Figure S10. HOR curves obtained on Pt(110) in 0.1M (a) LiOH, (b)NaOH, and (c) KOH, as well as 0.001 M (d) LiOH, (e) NaOH, and (f) KOH for different concentrations of corresponding $MeLiClO_4$ (5 mM, 20 mM, 50 mM and 100mM), where Me is Li, Na, or K, as indicated.

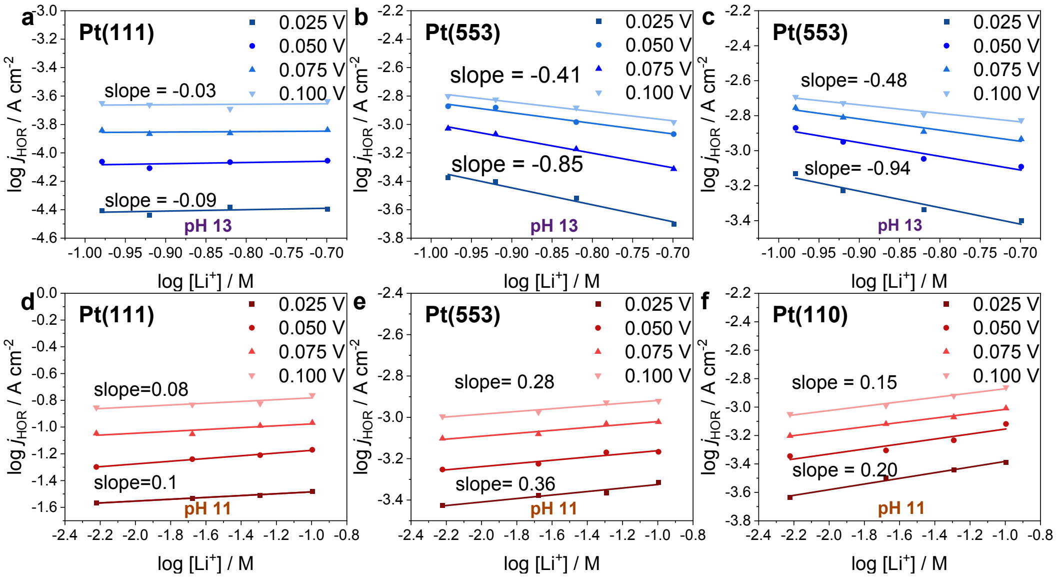

Figure S11. Reaction order plots obtained for HOR on (a) Pt(111), (b) Pt(553), and (c) Pt(110) at pH 13 and at pH 11 in (d) Pt(111), (e) Pt(553), and (f) Pt(110) in LiOH with varying cation concentration in the bulk at different 1600 rpm at a scan rate of 10 mVs^{-1} where the slope indicates the corresponding reaction order at a fixed potential (vs RHE).

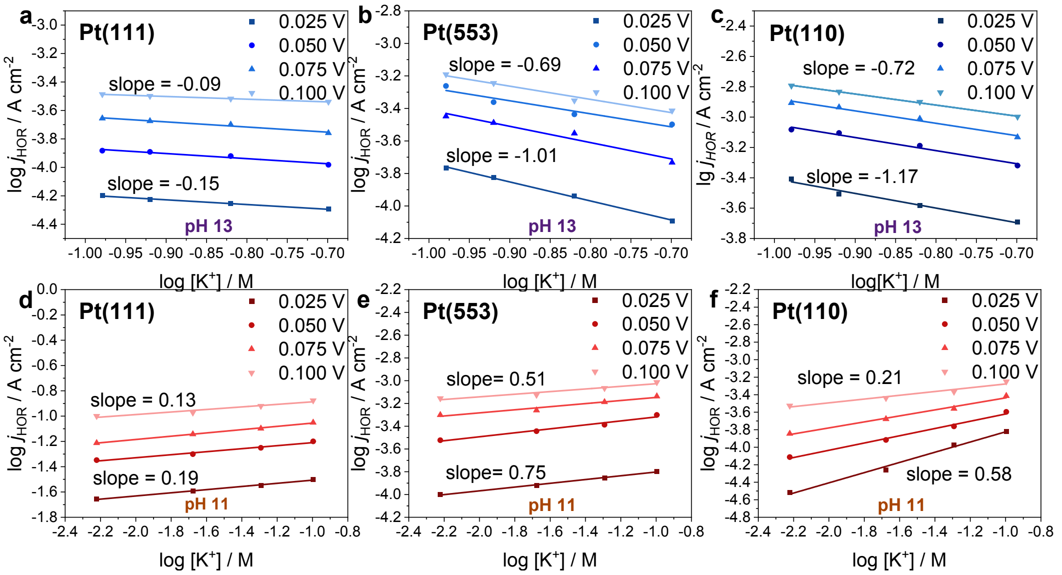

Figure S12. Reaction order plots obtained for HOR on (a) Pt(111), (b) Pt(553), and (c) Pt(110) at pH 13 and at pH 11 in (d) Pt(111), (e) Pt(553), and (f) Pt(110) in KOH with varying cation concentration in the bulk at different 1600 rpm at a scan rate of 10 mVs^{-1} where the slope indicates the corresponding reaction order at a fixed potential (vs RHE).
